# Inhibition of p300 lysine acetyltransferase activity by luteolin reduces tumor growth in head and neck squamous cell carcinoma (HNSCC) xenograft mouse model

**DOI:** 10.18632/oncotarget.6245

**Published:** 2015-10-26

**Authors:** Ruthrotha B. Selvi, Amrutha Swaminathan, Snehajyoti Chatterjee, Muthu K. Shanmugam, Feng Li, Gowsica B. Ramakrishnan, Kodappully Sivaraman Siveen, Arunachalam Chinnathambi, M. Emam Zayed, Sulaiman Ali Alharbi, Jeelan Basha, Akshay Bhat, Madavan Vasudevan, Arunasalam Dharmarajan, Gautam Sethi, Tapas K. Kundu

**Affiliations:** ^1^ Transcription and Disease Laboratory, Molecular Biology and Genetics Unit, Jawaharlal Nehru Centre for Advanced Scientific Research, Jakkur P.O., Jakkur, Bangalore, India; ^2^ Department of Pharmacology, Yong Loo Lin School of Medicine, National University of Singapore, Singapore; ^3^ Department of Botany and Microbiology, College of Science, King Saud University, Riyadh, Kingdom of Saudi Arabia; ^4^ Bionivid Technology [P] Ltd, East of NGEF, Bangalore, India; ^5^ School of Biomedical Sciences, CHIRI Biosciences Research Precinct, Curtin University, Bentley, Western Australia, Australia

**Keywords:** flavonoids, miRNA, gene expression, cancer

## Abstract

Chromatin acetylation is attributed with distinct functional relevance with respect to gene expression in normal and diseased conditions thereby leading to a topical interest in the concept of epigenetic modulators and therapy. We report here the identification and characterization of the acetylation inhibitory potential of an important dietary flavonoid, luteolin. Luteolin was found to inhibit p300 acetyltransferase with competitive binding to the acetyl CoA binding site. Luteolin treatment in a xenografted tumor model of head and neck squamous cell carcinoma (HNSCC), led to a dramatic reduction in tumor growth within 4 weeks corresponding to a decrease in histone acetylation. Cells treated with luteolin exhibit cell cycle arrest and decreased cell migration. Luteolin treatment led to an alteration in gene expression and miRNA profile including up-regulation of p53 induced miR-195/215, let7C; potentially translating into a tumor suppressor function. It also led to down-regulation of oncomiRNAs such as miR-135a, thereby reflecting global changes in the microRNA network. Furthermore, a direct correlation between the inhibition of histone acetylation and gene expression was established using chromatin immunoprecipitation on promoters of differentially expressed genes. A network of dysregulated genes and miRNAs was mapped along with the gene ontology categories, and the effects of luteolin were observed to be potentially at multiple levels: at the level of gene expression, miRNA expression and miRNA processing.

## INTRODUCTION

Reversible histone acetylation has been now recognised as a key epigenetic modification for the regulation of gene expression. The enzymatic machinery involved in this process has also been shown to possess important regulatory roles in physiological and pathophysiological conditions and therefore viewed as attractive therapeutic target. Small molecule modulators of these enzymes have been identified and their therapeutic efficacies being tested both in pre-clinical and clinical studies. However, the existing inhibitors suffer from one or more of the following shortcomings of non-specific mode of action, decreased cell permeability or metabolic instability. Hence, there is an active search for better inhibitors from natural sources as well as for the synthesis of better molecules. Surprisingly, a vast majority of the inhibitors have been identified from the natural sources and based on these scaffolds, efficient molecules have been synthesized (reviewed in [1] and references thereof). The first natural and potent acetyltransferase inhibitor (KATi), anacardic acid was isolated from Cashew Nut Shell Liquid (CNSL) was found to be poorly permeable to cells. Garcinol, a polyisoprenylated benzophenone from the kokum fruit (*Garcinia indica*) also found later on as a potent KATi with a non-specific mode of action, was subsequently derivatized into LTK14, a p300 specific KATi with promising therapeutic potential [2]. One of the most important natural source derived KATi is CTK7A, a water soluble derivative of curcumin which exhibited tumour regression ability in oral cancer xenograft model system [3]. Although these natural source-derived and modified KATi and several other synthetic KATi such as lysyl CoA [4] and C646 [5] have shown tremendous potential in several preclinical studies, most of these are limited by their working concentrations or their apparent difficulties to be metabolized. All these shortcomings have resulted in an ongoing effort to identify better and efficient molecules with improved therapeutic index.

During one such screening process, we had earlier identified that the crude extract of pomegranate fruit rind had a potent p300 acetyltransferase inhibitory activity. The subsequent purification of this extract had led to the identification of another active component which incidentally was an inhibitor of arginine methyltransferase [6], which was later used as a tool to understand the role of PRMT4 in neural/glial differentiation [7]. However, to find the potential new inhibitor in the crude extract, we decided to assay with the known components of the crude extract. Incidentally, majority of them were flavonoids, an important dietary constituent found in several edible fruits and vegetables. Flavonoids have been attributed with antioxidant property, potential to slow ageing, protection against cardiac diseases, ability to lower the risk of diabetes, cancer, stroke and also neurodegenerative disorders like dementia, Alzheimers' and Parkinsons'[8-10]. A major contribution to most of these diseases is ROS and the flavonoids have been considered to have protective effect against ROS generation [11]. Several efforts have been taken to identify the mechanism of action of these important dietary constituents and a few studies have reported their perturbations of the signalling pathways [12]. However, no studies have as yet reported any effect of the flavonoids on histone acetylation. Hence, we attempted to identify the possible mechanism of action of flavonoids in the context of epigenetic modifications with special emphasis on histone acetylation.

By employing systematic screening of the pomegranate skin tannins and flavonoids, we found that luteolin, but not the structurally related apigenin, is a potent, specific competitive inhibitor of p300 acetyltransferase activity with an IC50 of 7μM. Luteolin could inhibit histone acetylation in cultured cells and also in in vivo xenograft mouse model, the growth of which was retarded upon luteolin treatment. Apart from the direct effect on histone acetylation in the protein coding gene promoters resulting in downregulation of important cytokines like IL6, luteolin treatment also led to a significant alteration in the expression of miRNAs. Alteration of miRNA expression levels regulates the protein coding genes post transcriptionally. Interestingly, the profile of the upregulated miRNAs clustered towards tumor suppression, with many miRNAs being known inducers of cell cyle arrest and negative regulators of cell migration. The miRNAs with downregulated expression were predominantly classified into the oncomiR category. DICER, a component of the miRNA processing machinery was also found to be differentially expressed, which can also contribute to changes in miRNA processing. Thus, the important dietary flavonoid, luteolin was found to be a p300 acetyltransferase inhibitor, with the ability to modulate gene expression, miRNA expression and potentially, miRNA processing, correlating to a tumour suppressor outcome, which was also reflected in HNSCC tumor xenograft model.

## RESULTS

### Luteolin from PCE inhibits p300 KAT activity

**Figure 1:**
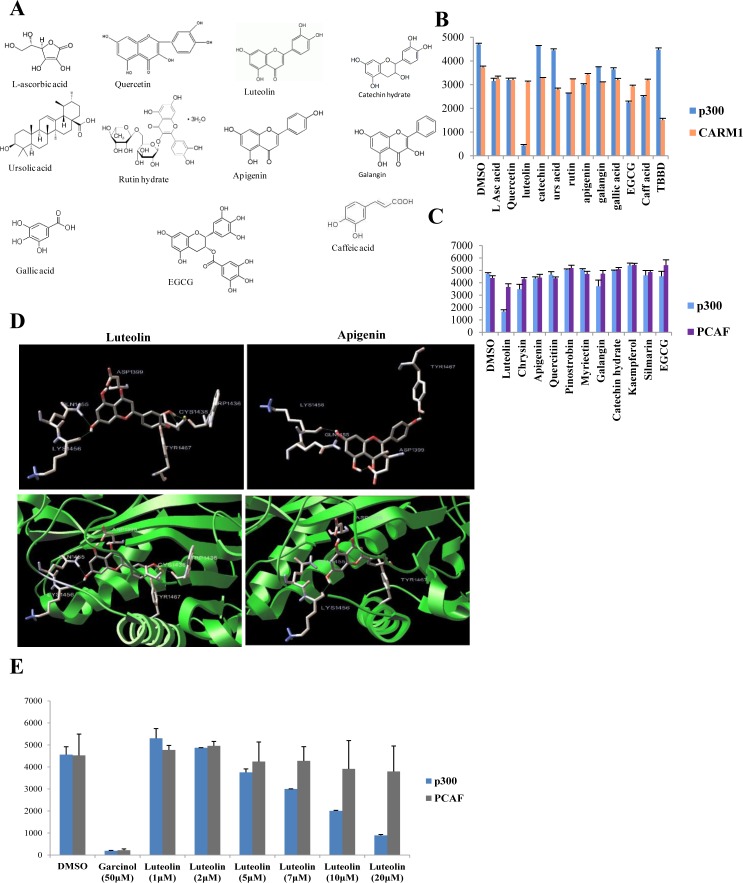
Luteolin is a p300 acetyltransferase inhibitor **A.** Structures of different components of the pomegranate crude extract. B, C, E. Filter binding assay was done with HeLa core histones as substrate using either hexahistidine tagged full length p300/FLAG tagged full length CARM1/FLAG tagged full length PCAF purified from recombinant baculovirus infected Sf21 cells. The components of the pomegranate crude extract (PCE) were assayed at a concentration of 50 μM in replicates **B, C.** TBBD (ellagic acid), a specific inhibitor of CARM1 and garcinol, an inhibitor of p300 were used as controls. Following the initial screen (**B**., **C**.), increasing concentration of luteolin was assayed in replicates to assess the effect on p300 and PCAF activity with HeLa core histones as substrate. **D.** Molecular docking studies of luteolin with p300 acetyltransferase domain depict key interactions within the acetyl CoA binding site. The structurally related apigenin which is deficient of the 3′ OH group in comparison with luteolin shows less favourable interaction.

The Pomegranate crude extract (PCE) components (Figure 1A) were screened using p300 acetyltransferase and arginine methyltransferase (RMTase), CARM1 (Figure 1B). Our earlier study had shown that the crude extract had a strong inhibitory activity on these two classes of enzymes [2]. Of all the compounds, only ellagic acid was found to inhibit CARM1 (Figure 1B). However, most strikingly luteolin could almost completely inhibit the acetyltransferase activity of p300 very potently while all the other components were ineffective.

Luteolin is a dietary flavonoid, which possesses diverse important physiological effects on human cells. The flavonoids include flavonols, dihydroflavonols, flavones, isoflavones, flavanones, anthocyanins, and anthocyanidins. Significantly, of the different flavonoids, we screened for the major category of flavones (luteolin, apigenin, chrysin, pinostrobin), flavonols (galangin, kaempferol, quercetin, myricetin, rutin, silymarin) and flavanols (catechin, EGCG), only luteolin showed potential p300 (but not PCAF) KAT inhibition ability (Figure 1C). These compounds are characterised by poly-hydroxy functional groups (Figure 1A). An earlier study from our group has shown that the -OH group is critical for the inhibition of KAT activity [13]. It is pertinent to point out here that even apigenin which structurally differs with luteolin only by one -OH group at 4′-position, could not affect the p300 or PCAF KAT activity (Figure 1C). Collectively, among all twelve flavonoids tested, only luteolin could inhibit the KAT activity of p300.

It was intriguing that luteolin could inhibit p300 whereas the structurally similar apigenin did not have any effect. To investigate whether the presence of an additional –OH group conferred luteolin with differential binding abilities; we performed a molecular simulation and docking study on p300 minimal HAT domain (Figure 1D). We identified that luteolin and apigenin had common binding sites on the acetyltransferase domain, of which the important one being Y1467-identified in the C646, p300 KAT inhibitor study [5]. However, the significant difference between luteolin and apigenin binding to the p300 HAT domain (HD) is with respect to two residues (W1436 and C1438). Luteolin exhibited binding to W1436 which is a critical residue on p300 HD. In agreement with this observation, point mutation of W1436 to alanine was found to abolish the KAT activity of the p300 HD in an earlier study [14]. Another unique residue for luteolin binding is C1438 which was identified as a part of the substrate binding loop L1 (Figure 1D). Interestingly, various cancers and Rubinstein Taybi syndrome is associated with mutations at C1438 [14]. The presence of an additional –OH group in luteolin led to the binding to two important residues on the p300 KAT domain which results in an apparent decrease in the inhibition constant by more than threefold as represented in supplementary table 1. On the other hand, the same analysis was also performed on PCAF acetyltransferase domain and although there were common binding sides, the observed Ki was too high which also correlated to the non-inhibitory pattern observed in the *in vitro* assays (Supplementary table 1).

As indicated in Figure 1E, luteolin inhibits p300 KAT activity with an IC_50_ of 7μM but had minimal effect on PCAF even at 20μM concentration. To elucidate the mechanism of inhibition of p300 activity by luteolin, kinetic analysis for the enzyme inhibition was performed. Luteolin inhibits p300 activity in competitive mode with acetyl-coA binding site, whereas it was observed to have characteristics of mixed inhibition with a predominance of competitive binding to the histone binding site (Supplementary figure 1).

These findings clearly indicate that luteolin is a potent acetyltransferase inhibitor with preferential specificity towards p300.

### Luteolin inhibits tumour progression by inhibiting histone acetylation in oral cancer cells and tumour xenograft

**Figure 2:**
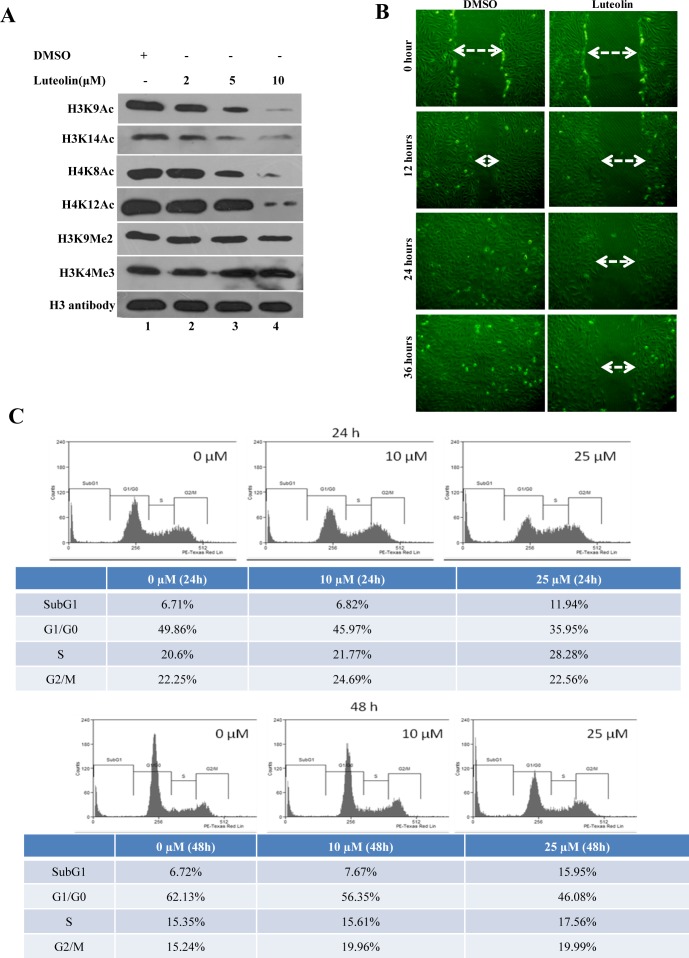
Luteolin mediated p300 acetyltransferase inhibition influences cell migration and cell proliferation **A.** KB cells were treated with luteolin at different concentrations and the status of histone modifications were analysed. Lane 1 represents histones isolated from the solvent control treated cells, whereas lanes 2-4 represent histones isolated from cells treated with 2, 5 and 10 μM of luteolin respectively. Histones were probed with different antibodies as indicated, with histone H3 used as loading control. **B.** UPCI:SCC029B oral cancer cells with wounds of constant diameter were treated with DMSO/luteolin (10μM) for 24 hr along with 10% serum. The wound photographs were taken under phase-contrast microscope. **C.** CAL27 cells (5×10^5^/ml) were treated with 10 μM and 25 μM luteolin for 24 and 48 hr respectively, after which the cells were washed, fixed, stained with PI, and analyzed for DNA content by flow cytometry.

**Figure 3:**
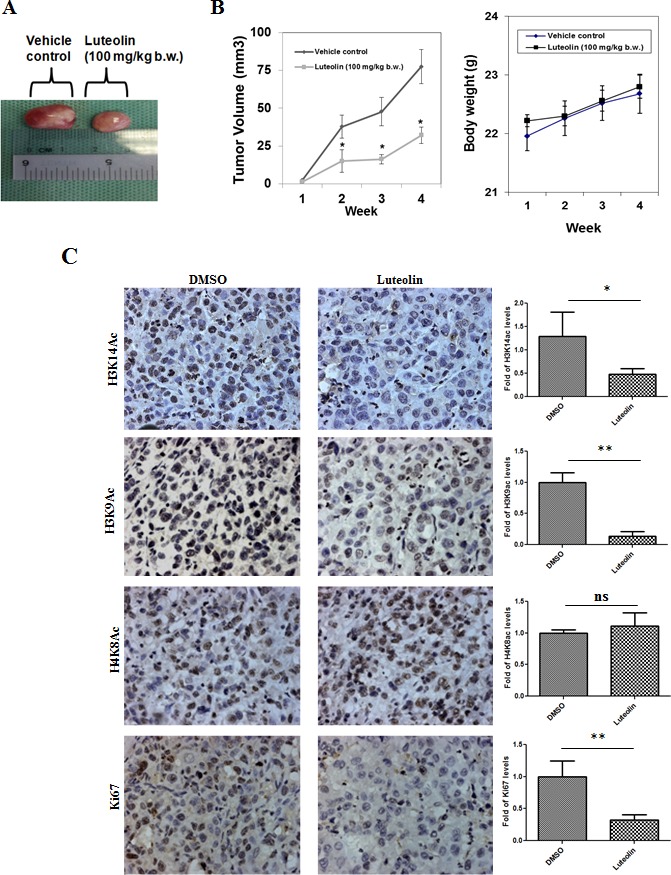
Histone acetylation inhibitor luteolin has *in vivo* anti-tumor activity **A.** Representative image of xenografted tumor of CAL27 cells in athymic balb/c nude male mice. **B.** Effect of luteolin (100 mg/kg b.w. administered i.p. for 4 week, five times per week) on body weight of mice. The data is represented as mean body weight in gm ± SE. **C.** IHC images of CAL27 derived tumor samples from mice treated with DMSO or luteolin, stained for H3K14ac, H3K9ac, H4K8ac and proliferation marker Ki67. The quantification depicts the decrease in the level of acetylation as well as proliferation status in the luteolin treated samples. Data is represented as mean tumor volume ± SE. *p < 0.05 for control group versus luteolin treatment group at the end of 4 weeks treatment.

To determine the physiological role for this inhibitor, KB cells which exhibit hyperacetylation were treated with luteolin. The acetylation inhibition was observed at low micromolar concentrations (5μM) in the oral cancer cell line after 6 hours of treatment, lower than the observed IC_50_ from *in vitro* assay. A decrease in histone H3 acetylation (H3K9 and H3K14) was observed upon luteolin treatment. At 10 μM concentration, an almost complete inhibition of these marks could be observed (Figure 2A, Lane 4). However, acetylation of histone H4 was relatively less inhibited and histone methylation was unaffected. These results show that luteolin is a potent inhibitor of KAT activity even in the cellular system at low concentration. Hyperacetylation of histones and nonhistone proteins are associated with the progression of oral and liver cancer [3, 15, 16]. Inhibitors of KATs have been implicated as one of the possible epigenetic therapeutics [1]. We decided to investigate the effect of luteolin on two important aspects of tumor progression; cell migration and cell proliferation. It was observed that treatment of luteolin to the UPCI:SCC029B oral cancer cells significantly reduced the wound healing ability in a dose dependent manner (Figure 2B). We also analyzed the effect of luteolin on cell cycle distribution in HNSCC cells and observed that after 24 h treatment with 25 μM luteolin, increased accumulation of cells were observed in S phase, which is indicative of cell cycle arrest. On the contrary, upon treatment with 25 μM luteolin for 48 h, around 16% of the cell population had accumulated in sub-G1 phase, which is indicative of apoptosis (Figure 2C). Taken together, these data suggest that luteolin acts as an anti-proliferative agent in oral squamous cancer cells in culture. In order to see if the same effect of luteolin was also observed *in vivo*, we analyzed whether luteolin can inhibit the growth of CAL27 xenografts in nude mice. We found that luteolin treatment (100 mg/kg body weight) administered intraperitoneally (i.p.) for 4 weeks, significantly suppressed the tumour growth *in vivo* following 4 weeks of treatment (Figure 3A and 3B). No significant change in body weight of luteolin treated mice was observed during the treatment, thereby indicating that luteolin is apparently non-toxic (Figure 3B, right panel). The alteration of histone acetylation levels was determined by performing immunohistochemical analysis of the xenografted mice tumours. Luteolin treated mice tumours showed decreased levels of H3K9 and K14 acetylation (Figure 3C) thus implicating its KAT inhibitory activity in the tumour tissue. Luteolin inhibited H3 acetylation more potently than H4 acetylation (Figure 3C) which is in accordance with the inhibition pattern from KB cells. The tumour samples also showed decreased levels of proliferation marker Ki67 in the luteolin treated mice. Reduced levels of Ki67 in the tumour sample support the anti-proliferation and anti-tumour activity of luteolin.

### Luteolin alters global mRNA and miRNA expression

**Figure 4:**
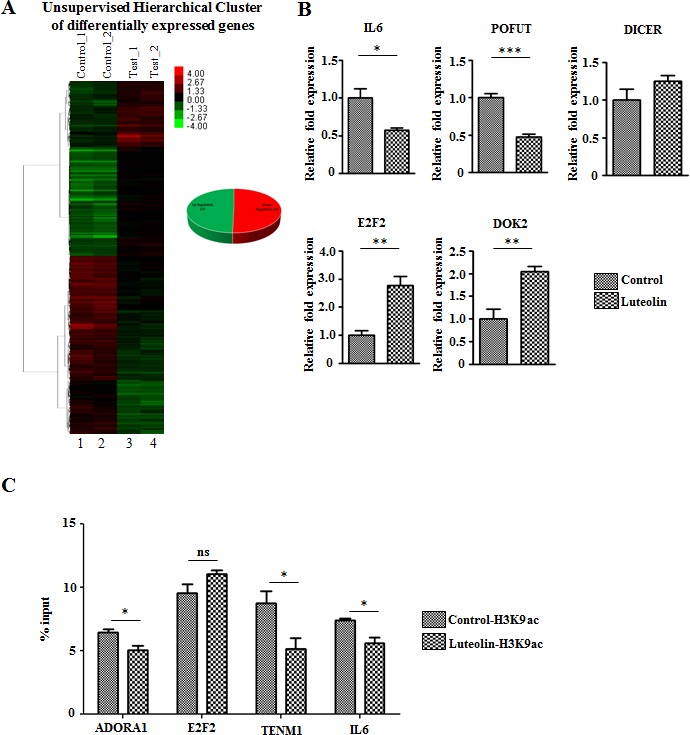
Luteolin mediated p300 acetyltransferase inhibition leads to an alteration in gene expression profile **A.** RNA from CAL27 cells treated with 10 μM of luteolin was subjected to whole genome microarray analysis to determine the changes in the gene expression profile. Heat map depicting the differentially expressed genes (2 fold and above with a *p* Value of <0.05) upon luteolin treatment (lanes 3 and 4) in comparison to control samples (lanes 1 and 2), in which equal number of genes was up- and downregulated. **B.** qPCR validation of representative differentially expressed genes closely related to tumor progression. (*n* = 3; *,*p* < 0. 05; **,*p* < 0. 01, ***, *p <* 0.001). **C.** Chromatin immunoprecipitation followed by qPCR performed with UPCI:SCC029B oral carcinoma cells treated with 10 μM luteolin for 8 hours. Quantification of immunoprecipitated DNA is represented relative to the input DNA. (*n* = 3; *p < 0. 05; ns, not significant). H3K9ac was normalized against H3 levels in the selected promoters in control DMSO treated and luteolin treated cells.

Since histone acetylation modulates gene expression, we investigated the effect of acetylation directly upon inhibition of p300 KAT activity in CAL27 cells. An equal number of genes were seen to be up- and downregulated upon luteolin treatment (Figure 4A). Upon closer inspection of the genes, genes like interleukin-6 (IL6), which are upregulated in other cancers [17-19] were seen to be downregulated upon luteolin treatment (Figure 4B). Also, genes like E2F2 which control the cell cycle [20, 21] and DOK2, the downregulation of which has been observed to worsen prognosis in multiple cancers [22-24], were upregulated (Figure 4B). These changes in the gene expression pattern suggested that luteolin causes changes in gene expression patterns favourable to inhibition of cancer progression. In order to understand the direct effect of luteolin mediated inhibition of histone acetylation on gene expression, we performed a chromatin immunoprecipitation analysis for H3K9 acetylation on gene promoters using UPCI:SCC029B oral cancer cell line treated with 10 μM luteolin. Interestingly, on the promoters of factors like IL6 and ADORA1, which are upregulated in cancers and are associated with poor prognosis, histone acetylation was found to be significantly repressed upon luteolin treatment (Figure 4C). In addition, luteolin treatment could significantly reduce the H3K9 acetylation on the promoter of TENM1, which is observed to be upregulated in several cancers. However, histone acetylation on the promoter of tumor suppressor E2F2 showed no significant change in response to luteolin. Thus, the inhibitory potential of luteolin could directly be correlated with reduced acetylation on gene promoters, especially in case of tumor promoting factors.

**Figure 5:**
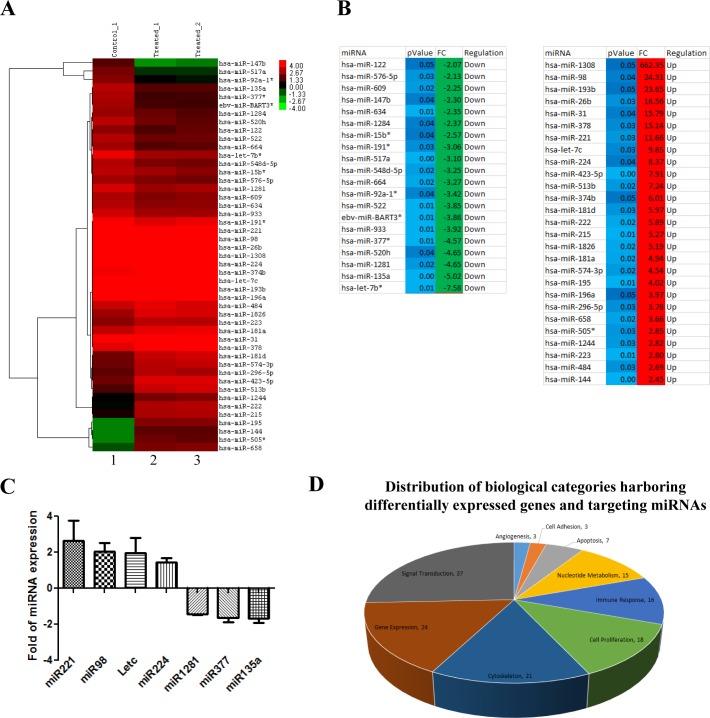

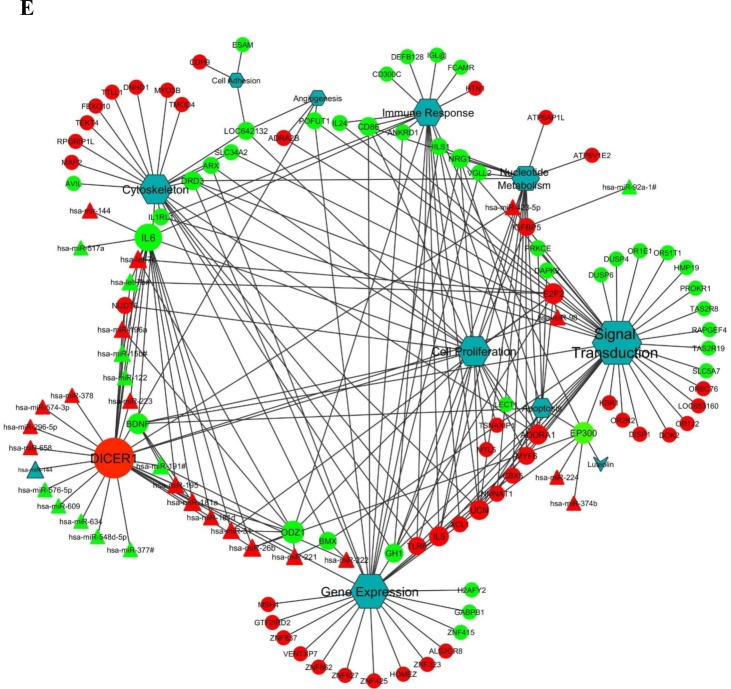
Luteolin mediated p300 acetyltransferase inhibition influences miRNA network correlating to tumor suppression **A.** RNA from KB cells treated with 10 μM of luteolin was subjected to whole genome miRNA microarray analysis to determine the changes in the miRNA profile. Heat map depicting the differentially expressed miRNA (2 fold and above with a *p* Value of <0.05) upon luteolin treatment (lanes 2 and 3) in comparison to untreated samples (lane 1). **B.** List of induced and repressed miRNA upon luteolin treatment with fold change of 2 and above with a *p* Value of <0.05 (calculated by Student T-test. **C.** qPCR validation of the selected differentially expressed miRNA upon treatment with 10μM luteolin for 6hrs in UPCI: SCC029B cells. **D.** Distribution of biological categories harboring differentially expressed genes and targeting miRNAs. **E.** miRNA perturbation and gene network upon luteolin treatment. The presence of cell proliferation in the middle of the network indicates significant dysregulation of genes closely associated with cancer. Also, the mapping of DICER as an important node suggests that the activity of luteolin is at multiple levels: at the level of gene expression, miRNA expression and miRNA processing.

Interestingly, we noted that DICER showed an upregulation of expression upon luteolin treatment (Figure 4B). This suggested possible changes in the miRNA profile in addition to the gene expression changes that were observed. Indeed, we observed that treatment of luteolin in KB cells triggered both up-regulation as well as down-regulation of various sets of miRNA (Figure 5A). About 40 miRNAs including let7c showed more than 2-fold differential expression in 10μM luteolin treated cells over DMSO treated cells (Figure 5B). The alteration of miRNA expression by luteolin was also validated in the UPCI:SCC029B oral squamous cell carcinoma cells. Treatment of luteolin induced upregulation of miR221, miR98 and let7c as well as downregulated expression of miR135a, miR1281 and miR377 were down-regulated as also validated by qRT-PCR analysis (Figure 5C). A large percentage of the dysregulated genes and miRNAs belonged to the categories of gene expression, signal transduction and cell proliferation, which are the principal processes controlling cancer progression (Figure 5D).

Interestingly, luteolin has been shown to downregulate cell cycle associated genes in several cancer cell models and partly has been attributed to its effect on the phospho-signaling cascade albeit in higher concentrations than reported here [25]. Hence, in order to visualize the interactions that are associated with p300 acetylation inhibition by luteolin with gene and miRNA perturbations, we modelled a miRNA and gene network map. This analysis clearly portrays a complex regulatory network with various genes and miRNAs centered around cell proliferation and signal transduction (Figure 5E). As predicted earlier, DICER forms an important node, indicating that luteolin influences miRNA expression and miRNA processing in addition to the other effect on other pathways. Also, factors like IL6, ADORA1, ODZ1 and E2F2 which have been validated by H3K9ac ChIP, formed important nodes in this network.

Taking together all these observations, it is clear that luteolin treatment leads to an overall perturbation of gene expression and miRNA profile favouring a tumor suppression effect, thereby signifying the protective role of the dietary flavonoid luteolin as an anticancer agent.

## DISCUSSION

Crude extract of pomegranate (PCE) has been considered as a therapeutic agent for several decades and its medicinal values have been observed as anti-microbial, anti-parasitic, anti-viral and anti-cancer effects. Ellagic acid or TBBD isolated from PCE exerts PRMT4/CARM1 inhibitory effect which specifically translates to histone H3R17 methylation inhibition without affecting H3R26 methylation [6]. In our present study, we observed that a few components of PCE also inhibit lysine acetyltransferase activity of p300. A few of these, such as gallic acid and EGCG have already been reported to have epigenetic modulatory effect [26, 27]. A significant observation from our study was that an important dietary flavonoid, luteolin was found to be a potent p300 acetyltransferase inhibitor. Apart from the reported EGCG and luteolin (identified in our present study), no other component exhibited any significant p300 inhibition. The flavanoids and luteolin are widely acclaimed for their antioxidant properties and their overall protective effect. The fact that they are present in edible fruits and vegetables, implicates their biological utility. We also identified that the structurally related apigenin does not inhibit p300 activity which could be ascribed to their differential binding on to the p300 HAT domain. A significant observation is that when apigenin is tested *ex vivo* on tissues, it is known to get converted to luteolin and then metabolised by the system [28]. All these studies suggest that luteolin is a biologically useful molecule and it can be metabolised without any accumulation and downstream side effects. Several theories have been presented to elucidate the mechanism behind the anticancer activity of luteolin. Recently, it was reported that luteolin potentiates histone deacetylase inhibition activity [29] whereas another report suggests that it inhibits kinase activity of aurora B [30] and thereby blocks cancer cell proliferation. However, it inhibits HDACs at a very high concentration (100μM) as compared to its kinase and acetylation inhibitory concentration (5-10μM). Histone hyperacetylation is a common characteristic of oral cancers and thus the KAT inhibitors have proved to be a promising therapeutic agent against it. Luteolin inhibits tumour progression by inhibiting angiogenesis probably by inhibiting acetylation, as p300 acetyltransferase activity is closely related to angiogenesis. Silencing p300/CBP results in compromised tube formation and inhibits angiogenesis [31]. Several studies on cancer models both as cell lines and tumours have investigated the effect of luteolin on angiogenesis and it has been shown that luteolin downregulates VEGF expression [32]. A major conclusion from our study also supports the antiangiogenic property of luteolin. There is also evidence of luteolin mediated induction of apoptosis in the oral cancer cell line used for the xenograft study as observed by the cell cycle analysis. We show that luteolin inhibits acetylation in cells, as well as in HNSCC xenograft tumor model. Histone acetylation regulates gene expression, and treatment with luteolin resulted in dysregulated expression of important inflammatory cytokines and cell cycle regulatory proteins, this being a direct downstream effect of reduced histone acetylation on these gene promoters. Consistently, gene expression, cell proliferation and immune response formed important nodes when we mapped the gene expression and miRNA expression data as a network. The GO clustering of the altered miRNA clearly implicate an upregulation of tumor suppressive miRNA and a down-regulation of tumorogenic miRNA expression. Although broad spectrum HDACi treatments alter the miRNA expression profile, the direct role of KAT activity in miRNA expression is yet to be elucidated. Significantly, we found that one of the upregulated genes, DICER formed an important node, indicating potential regulation of miRNA maturation, along with changes in expression of miRNAs. Knocking down DICER leads to changes in chromatin signature and hence, affects gene expression, with an increase in histone acetylation and decrease in methylation [33-35]. Hence, the changes in gene and miRNA expression might also be a result of other additional phenomena, apart from the direct effect of luteolin on p300-mediated histone acetylation.

We found that 6 hours treatment of luteolin to KB and SCC029B cells differentially regulates the expression of miRNAs which play crucial roles in angiogenesis and cancer cell proliferation. Incidentally, the pomegranate juice components tested against prostate cancer cells also revealed a similar miRNA alteration profile [36]. However, in this report we have provided a concise miRNA-gene network that is exclusive to luteolin treatment at an IC50 of 7μM concentration in the oral cancer cells. This finding may contribute immensely to our search for a therapeutic agent which is obtained from natural products that may prove to be valuable tool to fight against oral cancer. This study also appropriately connects the alteration of histone acetylation to tumour regression through regulation of gene expression directly and by miRNA regulation.

## MATERIALS AND METHODS

### Filter binding assay

KAT/RMT assays were performed as described previously [13] and are described in the supplementary section.

### Immunoblotting assay

KB cells (1.5 × 10^6^ cells per 60-mm dish) were seeded overnight, and histones were extracted by acid extraction after 6 hours of treatment with increasing concentrations of luteolin. Immunoblotting analysis was performed as described elsewhere [13], using polyclonal acetylated histone H3K9, H3K14, H4K8, H4K12, H3K9me2, H3K4me3 and polyclonal histone H3 antibodies.

### Molecular docking studies

Crystal structure of p300 KAT domain was extracted from Protein Data Bank code 3BIY. Crystal structure of the luteolin and apigenin was obtained and solved (Bruker X8 APEX). The KAT domain was docked with the structure of luteolin and apigenin to find out their interaction sites on KAT domain.

### HNSCC cancer xenograft mouse

Model was used as described in [37]. Control group was treated with 0.1% DMSO and treatment group mice received intraperitoneal injection (i.p.) 100 mg/kg luteolin five times a week for 4 weeks from the date of randomization.

### Immunohistochemical analysis of tumor samples

Was performed as described in [37] and is described in the supplementary section.

### Gene expression analysis

Was performed as described earlier in [6]. Briefly, following treatment with luteolin, total RNA was isolated using TRIzol reagent (Invitrogen). cDNA was synthesized with oligo(dT) (28-mer) (Invitrogen) and Moloney murine leukemia virus reverse transcriptase (Sigma), and expression analysis was carried out using SYBR Green supermix (Kapa) and gene-specific primers.

### Microarray and miRNA array

Were performed using RNA extracted using the Trizol method. The array and the following analysis was performed as described previously [7] and is described in the supplementary section.

### miRNA expression analysis

Invitrogen Ncode VILO cDNA synthesis kit was used to make cDNA from RNA isolated by Trizol method, following the manufacturer's instructions. The cDNA made thus was used for downstream qRT-PCR based profiling.

### Wound healing assay

Was performed as described earlier in Arif M et al. [3].

### Flow cytometric analysis

To determine the effect of luteolin on the cell cycle, CAL27 cells (5×105/ml) were seeded in 6-well plate and then treated with to 10 μM and 25 μM luteolin for 24 and 48 h respectively. Thereafter cells were trypsinized, washed with PBS, and fixed with 70% ethanol for 30 min on ice. Cells were then washed again, resuspended, and stained in PBS containing 10 μg/ml propidium iodide (PI) and 1 μg/ml RNase A for 30 min at room temperature. Cell distribution across the cell cycle was analyzed with a CyAn ADP flow cytometer (Dako Cytomation).

Chromatin Immunoprecipitation analysis was performed as described in ref 6.

## SUPPLEMENTARY MATERIAL FIGURE AND TABLE


