# Supplementary material for: Inhibition of p300 lysine acetyltransferase activity by luteolin reduces tumor growth in head and neck squamous cell carcinoma (HNSCC) xenograft mouse model
Source: Oncotarget. 2015 Oct 26;6(41):43806–18. doi: 10.18632/oncotarget.6245 (PMC4791268; doi:10.18632/oncotarget.6245)
Supplement: Supplementary file 1 [file oncotarget-06-43806-s001.pdf]

Inhibition of p300 Lysine Acetyltransferase activity by Luteolin reduces tumor growth in head and neck squamous cell carcinoma (HNSCC) xenograft mouse models

Supplementary Material

Methods:

Filter binding assay:

2.4 µg of highly purified HeLa core histones were incubated in assay buffer at 30 °C for 10 min with or without baculovirus- expressed recombinant p300 or PCAF or CARM1 in the presence and absence of compounds followed by addition of 1.0µl of 3.6 Ci/mmol of [^{3}H]acetyl-CoA/ [^{3}H] SAM (PerkinElmer Life Sciences) and further incubated for another 10 min in a 30µl reaction. The reaction mixture was then blotted onto P81 (Whatman) filter paper and radioactive counts were recorded on a Wallac 1409 liquid scintillation counter.

Immunohistochemical analysis of tumor samples:

Solid tumors from DMSO and luteolin injected mice were fixed with 10% phosphate buffered formalin, processed and embedded in paraffin. 4 micron sections were cut and deparafinized in xylene, and dehydrated in graded alcohol and finally hydrated in water. Antigen retrieval was performed by boiling the slide in 10 mM sodium citrate (pH 6.0) for 30 min. Immunohistochemical analysis were performed using antibodies against histone H3K9ac , H3K14ac, H4K8ac, H4K12ac and proliferation marker Ki67 and developed by using 3, 3-diaminobenzidine tetrahydrochloride (DAB) as a substrate. Sections were counterstained with Gill's hematoxylin and mounted under glass cover slips. Images were taken using a Zeiss fluorescent microscope. Positive cells (brown) were quantified using the Fiji Image J software. Three samples for each treatment (3 tissues for control DMSO and 3 tissues for Luteolin treated tumors) and one slice per animals were used for quantification.

miRNA array and analysis: Approximately 100 nanograms of total RNA extracted from Luteolin (10 µM) treated and DMSO treated KB cells were labeled using the Agilent miRNA Complete Labeling and Hybridization Kit (Agilent Technologies INC, Santa Clara CA, USA) according to the manufacturer's instructions. The labeled RNA was hybridized to the Agilent Human miRNA Microarray (V3, Agilent) which contains probes for 866 mature human miRNAs. Arrays were scanned using an Agilent scanner and feature extracted using Agilent Feature Extraction Software, version 10.5.1.1. Expression data were initially normalized to the 90th percentile and then averaged among the replicate groups using GeneSpring GX v 12.0 (Agilent Technologies INC, Santa Clara, CA, USA) software. Differentially expressed miRNA upon Luteolin treatment in comparison to DMSO treatment was identified using Volcano Plot method. This method applies Student-T-test and the Benjamini-Hochberg correction to identify statistically significance of the change. miRNAs that were 2 fold and above increased or decreased upon Luteolin treatment in comparison to DMSO were considered as differentially expressed. Unsupervised hierarchical clustering of differentially expressed miRNA was done using Pearson Uncentered algorithm with average linkage rule. MicroCosmTargetsversion-5[http://www.ebi.ac.uk/enright-srv/microcosm/htdocs/targets/v5/] was used to identify the gene targets of differentially expressed miRNAs. Genes targeted by differentially expressed miRNA was queried using GoElite tool (www.genmapp.org/go_elite/) to identify significant biologcial processes, pathways and functions. miRNA-Gene-Biological process regulatory network modeling was done using Cytoscape V 8.0.

Inhibition Kinetics: The KAT reactions were carried out with the baculovirus expressed recombinant full-length p300, in the presence of different concentrations of luteolin (5 and 10 µM). The KAT reaction consists of two substrates, core histones and the acetyl group donor [^{3}H] acetyl-CoA. Therefore, the kinetic analysis was done in two different sets. In the first

set, concentration of core histones was kept constant at 1.6 M and [3H] acetyl-CoA was varied from 1.08 to 8.66 M. In the second assay, [3H] acetyl-CoA was kept constant at 2.78M, and core histones were varied from 0.003 to 0.068 M. The incorporation of the radioactivity was taken as a measure of the reaction velocity recorded as counts/min. Each experiment was performed in triplicate, and the reproducibility was found to be within 15% of the error range. Weighted averages of the values obtained were plotted as a Lineweaver-Burk plot using Graph-Pad Prism software.

Primer information:

qRT-PCR primers:

Gene ID	Forward primer (5’-3’)	Reverse primer (5’-3’)
IL6	ACTCACCTCTTCAGAACGAATTG	CCATCTTTGGAAGGTTCAGGTTG
POFUT1	AACCAGGCCGATCACTTCTTG	GTTGGTGAAAGGAGGCTTGTG
E2F2	CCAGATCCAGCTTCCTTTTG	CTACACACCGCTGTACCCG
DOK2	CGGCCTGATAGCCCCTACT	GCTGTCGTACAGAGGGTCG
DICER	AAAATTGTCCATCATGTCCTCGC	CCACCAGGTCAGTTGCAGTT

To assess miRNA expression levels, a universal primer was used in combination with an miRNA sequence specific primer corresponding to the miRNA sequence itself.

ChIP qPCR primers:

Gene ID	Forward primer (5’-3’)	Reverse primer (5’-3’)
IL6	CGACGGCATCTCAGCCCTG	CAGAAGTCAGTCTAGTCGGCCC
TENM1	CCGCTGCCAGAAGGGTGCTTG	GCACCCGGGAGCGCGGCGAG
E2F2	ATGAATGGCGGCAACGCTGG	CAGGACCCAGTGCCCCCAAT
ADORA1	GGATGCGCCTCTGTCCAGTCC	CAATCGGGCTCCGCACACC

Supplementary data:

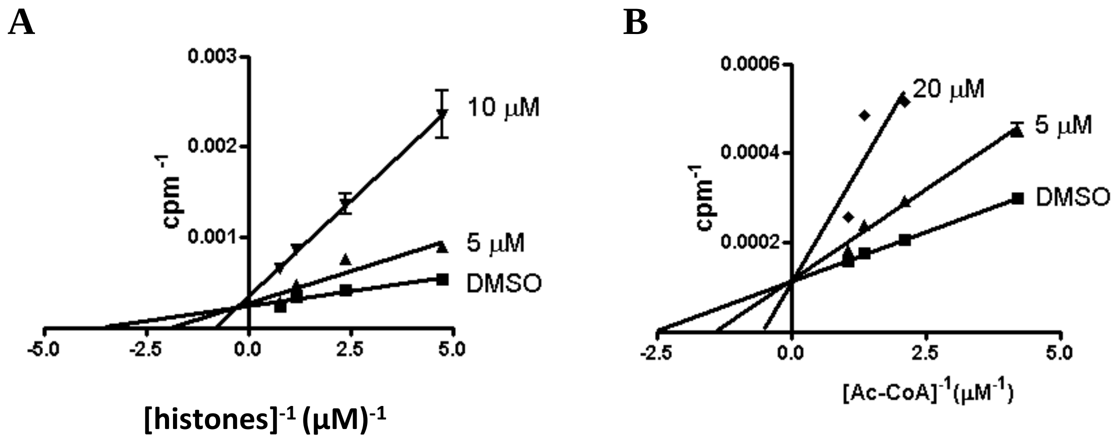

Supplementary Figure 1

A, B. Inhibition kinetics of luteolin was done using HeLa core histones as substrate and full length p300, with two different concentrations of luteolin (5 and 10 µM). The luteolin mediated inhibition was found to be competitive for the acetyl-CoA binding site (B) whereas it was found to have characteristics of mixed inhibition with a predominance of competitive binding to the histone binding site (A).

Supplementary table 1: Binding sites and parameters such as docking energy, inhibition constant obtained from the molecular docking study of luteolin with p300 and PCAF acetyltransferase domain

Molecular docking studies of luteolin with the p300 acetyltransferase domain and PCAF acetyltransferase domain

With p300

LIGAND NAME	COMMON BINDING SITES SHOWING H-BONDS	OTHER BINDING SITES SHOWING H-BONDS	DOCKING ENERGY	INHIBITION CONSTANT	TORSIONAL ENERGY
APIGENIN	Asp 1399<br>Gln 1455<br>Lys 1456<br>Tyr 1467	None	-8.45 kcal	636.99 nM	1.19 kcal
LUTEOLIN	Asp 1399<br>Gln 1455<br>Lys 1456<br>Tyr 1467	Cys 1438<br>Trp 1436	-9.06 kcal	229.18 nM	1.49 kcal

With PCAF

LIGAND NAME	COMMON BINDING SITES SHOWING H-BONDS	OTHER BINDING SITES SHOWING H-BONDS	DOCKING ENERGY	INHIBITION CONSTANT	TORSIONAL ENERGY
APIGENIN	Cys 559<br>Val 572	Gly 586	-235.75 kcal	414 µM	1.19 kcal
LUTEOLIN	Cys 559<br>Val 572	Thr 569<br>Tyr 616	-393.69 kcal	221 µM	1.49 kcal

C559-

V 572- part of the cleft meant for lysine substrate

G586- interaction with the pantetheine arm as well as pyrophosphate group of CoA (Direct and water mediated hydrogen bonds 582-587)

T569-

Y616- interacts with the pantetheine arm of CoA (A613, Y616, F617 involved in Van der Waal’s contacts, helping in orienting the sulfhydryl group for acetyl transfer, Y616 shows additional VDW interactions)
